# Special Issue “Chronic Fatigue Syndrome/Myalgic Encephalomyelitis: Diagnosis and Treatment”

**DOI:** 10.3390/jcm11154563

**Published:** 2022-08-04

**Authors:** Lorenzo Lorusso, Giovanni Ricevuti

**Affiliations:** 1 Neuroloy and Stroke Unit, Neuroscience Department, A.S.S.T.-Lecco, 23807 Merate, Italy; 2 Department of Drug Sciences, School of Pharmacy, University of Pavia, 27100 Pavia, Italy

Chronic fatigue syndrome, or myalgic encephalomyelitis (CFS/ME), is a debilitating disease with unknown causes that is more common in women and tends to develop between patients’ mid-20s and mid-40s. From the perspectives on the etiology and pathophysiology, CFS/ME has been labeled differently, which has influenced changes in case definitions and terminologies. CFS/ME is characterized by persistent asthenia with associated musculoskeletal pain, cognitive disturbance (including attention, memory, and concentration), psychological troubles (depression, anxiety), sleep disorders, and a variety of neurovegetative symptoms. The best appropriate therapeutic is an integrative approach, based on a personalized medical plane that includes distinct groups of procedures: educational, cognitive-behavioral, pharmacological and non-pharmacological such as occupational therapy and rehabilitation. CFS/ME has some common clinical features with fibromyalgia, and a differential diagnosis is difficult for General Practitioners (GPs) [1,2].

The recent opinion is that CFS/ME pathogenesis is dependent on several factors or causes. Different studies have shown evidence for an alteration in immunity system in patients with CFS/ME. A modification in cytokine subsets, a diminished activity of natural killer (NK) lymphocytes, the detection of autoantibodies and a decreased response of T cells to mitogens and specific antigens have been observed. An increased level of pro-inflammatory cytokines may explain some of the clinical features, such as fatigue and flulike symptoms, with an effect on NK activity. Anomalous activation of the T lymphocyte profile and a reduction in antibody-dependent cell-mediated cytotoxicity have been reported. An increased number of CD8+ cytotoxic T lymphocytes and CD38 and HLA-DR activation markers have been demonstrated, and a reduced CD11b expression associated with an increased expression of CD28+ T subsets has been described [3]. An interest towards CFS/ME is increased with the recent pandemic by SARS-CoV-2 because, after the acute phase of disease, some patients have clinical features similar to CFS/ME called Long-COVID, characterized by tiredness, brain fog and headache. There is debate on common aspect between these pathologies but in especially a possible effect of COVID-19 on CFS/ME and the consequences [4].

This Special Issue on CFS/ME collects 18 papers with an interdisciplinary view on the current demographic and epidemiological data and immunological characteristics of CFS/ME and examines the different pathogenic hypotheses, as well as giving information about the latest knowledge on diagnostic investigations, pharmacological, integrative, physical, cognitive-behavioral and psychological curative approaches.

It is known that CFS/ME affects young adults, but there are little studies in pediatric and adolescent age. Australian colleagues Elisha K. Josev and colleagues have carried out a case-controlled follow-up study on the health, wellbeing and prognosis of Australian adolescents with CFS/ME on the comprehension of the important relation between physical and psychological health factors to adolescent’ long-term outcome for approaching future prevention, management and treatment [5]. Concerning epidemiological data, there is little information for Asian countries such Korea and Japan. Eun-Jn Lim and Chang-Gue Son evaluate and match the prevalence of CFS/ME in Korea and Japan, performing a meta-analysis analyzing the main characteristics of these nations [6]. The emerging data of the involvement of immune system confirmed the hypothesis that CFS/ME is an autoimmune disease; recent studies have shown the role of autoantibodies towards the vegetative nervous system. Freitag H. and colleagues reported the reactivity of autoantibodies to vasoregulative G-Protein-Coupled Receptor correlates with autonomic dysfunction, clinical gravity and disability in CFS/ME patients [7]. Another paper, by Kujawski S. and collaborators, studies the differences in CFS patients applying post-exertional malaise (PEM) as indicators of aortic stiffness, autonomic nervous system function and severity of fatigue [8]. Always on the role of the autonomic nervous system dysfunction, Jessica Van Oosterwijck et al. published a paper showing decreased parasympathetic reactivation from physical exercise that could be correlated with a bad prognosis or high risk for adverse cardiac event [8]. Varesi A. and colleagues investigated the emerging role of the modified composition of gut microbiota in relationship with genetic, infection, immunological and other influences that have seen in CFS/ME individuals [9]. The authors discuss the change and the potential therapeutic application of treating the gut in CFS/ME patients [10].

A collection of papers investigates the importance of the diagnostic tools in clinical practice. We start with Baklund H. I. et al., who evaluated the blood test in relationship with clinical features and diagnostic classification, suggesting muscle damage and metabolic abnormalities [11].

A potential blood diagnostic tool, by Castro-Marrero J. and his Spanish collaborators, could be the complement C1 examining in CFS/ME three-symptom clusters, identified as severe, moderate and mild, presenting important differences in five blood parameters [12]. Another objective measurement for PEM, which is a hallmark of CFS/ME, is the application of the two-days cardiopulmonary exercise test (CPET) to assess functional impairment: Eun-Jin Lim and Korean collaborators, in their paper, published the results of a meta-analysis on this diagnostic tool [13]. Moreover, Do-Young Kim and his Korean colleagues examined a systematic review to provide an overview of the adoption of the main measurements in RCTs for CFS/ME. Around 40% of RCTs utilized multiple primary measurements. This information could be helpful in clinical practice in the design of medical studies for CFS/ME-linked therapeutic development [14].

The therapy of CFS/ME is problematic due to lack of knowledge on the etiopathogenesis of this disease, with application of the unconventional and conventional treatments: Tirelli and colleagues compared the application of oxygen–ozone autohemotherapy (O_2_-O_3_-AHT) in male vs. female patients, evaluating the differences in their responses to this approach [15]. The effects of exercise from a structured activity program have been disputed; Kujawski S. et al., with a multidisciplinary study, examined the impact of a personalized program of activities associated with cardiovascular, mitochondrial and fatigue parameters, showing a reduction in fatigue and an improving functional performance [16]. An important conventional therapeutic approach is the effect of s.c. IgG self-treatment in ME/CFS patients with IgG/IgG subgroup deficiency. The aim of Scheibenbogen C. and her German collaborators was to study the IgG administration for its immunomodulatory effects. [17].

There are few studies relationship CFS/ME patients and COVID-19 patients [18]. Araja D. and Latvian collaborators researched undiagnosed CFS/ME patients, hypothesizing the expansion of post-viral CFS as an effect of COVID-19 and its social impact. The Latvian research results show that patients with CFS/ME are not a risk group for COVID-19; however, COVID-19 causes symptoms similar to CFS/ME. They concluded that CFS/ME creates a significant social consequence, considering the direct medical costs of undiagnosed patients. At the same time, COVID-19 is responsible for long-lasting complications and a chronic course, such as post-viral CFS [19].

Deumer U-S et al. discuss the role of the gut microbiota on disease progression, highlighting a potential biomarker in non-coding RNA (ncRNA) as a probable diagnostic tool and suggesting the possibility that SARS-CoV-2 infection may result in symptoms similar to CFS [20].

CFS/ME has an overlap with Fibromyalgia, and differential diagnosis is difficult for some clinicians because the diagnosis of fibromyalgia is based only on clinical features that are characterized by widespread pain, fatigue, stiffness and troubles in cognitive functions, such as attention, executive function and verbal memory deficits [21]. It is important to add more tests beyond the Mini-Mental State Examination (MMSE) and the Montreal Cognitive Assessment (MoCA) test in fibromyalgia patients to assess the relationship between physical and cognitive performance, as reported by Murillo-Garcia A. and colleagues [22]. Another potential diagnostic tool is studied by Martin-Brufau R. and collaborators using electroencephalography for patients with fibromyalgia that present lower levels of brain activity with reduced connectivity than controls. The Spanish group identified a possible neurophysiological pattern that could adapt to the clinical features of the disease [23]. The therapeutic approach to this disease is a difficult choice. Rodriguez-Mansilla J. and Spanish collaborators studied the effects of non-pharmacological treatment in terms of the effectiveness of an exercise program compared to wellness activities by improving pain, flexibility, static balance, perceived effort and quality of life in patients with fibromyalgia. Participants in the active exercise program performed better than exercise for well-being [24]. This proposal in fibromyalgia is associated with other conventional treatments based on a multidisciplinary approach.

In conclusion, the papers published within this research topic, with the major contribution of the members of the European Network on Myalgic Encephalomyelitis/Chronic Fatigue Syndrome (EUROMENE), give us the recent highlight perspective and opportunities for the discovery and development of possible specific biomarkers, diagnostic and therapeutic approaches for these immunological disorders.
